# GAME-BASED VISUAL FEEDBACK-GUIDED DYNAMIC BALANCE TRAINING VERSUS CONVENTIONAL TRAINING IN PATIENTS WITH HEMIPLEGIA: A PILOT RANDOMIZED CONTROLLED TRIAL

**DOI:** 10.2340/jrm.v57.41277

**Published:** 2025-02-12

**Authors:** Yan XU, Jiadong NI, Yajing YANG, Jiajia YAO, Lianhui FU, Cong XU

**Affiliations:** From the Department of Neurology and Neurological Rehabilitation, Yangzhi Rehabilitation Hospital (Shanghai Sunshine Rehabilitation Center), School of Medicine, Tongji University, Shanghai, China

**Keywords:** balance dysfunction, balance training, game, hemiplegia, visual feedback

## Abstract

**Objective:**

This study aimed to compare the efficacy of game-based visual feedback-guided dynamic balance training with conventional training in patients with hemiplegia.

**Design:**

Single-blind, pilot randomized controlled trial.

**Patients:**

Fifty-three patients with hemiplegia exhibiting balance dysfunction at ≥ 1 month were enrolled.

**Methods:**

The experimental group (*n* = 25) trained with the Physio Space™ system that uses a 3-axis balancing platform and touchscreen monitor to provide visual feedback during 9 game modules at varying challenge levels for 20 min per day, 5 days per week, for 3 weeks. The control group (*n* = 28) received traditional training for the same duration. To evaluate the efficacy of the training, a 2-way repeated-measures analysis of variance (ANOVA) was used. The study protocol was registered on the Chinese Clinical Trial Registry (ChiCTR2300067275, https://www.chictr.org.cn).

**Results:**

Eighteen participants in each group completed the training programme, with no baseline between-group differences. The post-training assessment revealed significant improvements (Berg Balance Scale, Functional Reaching Test, Timed “Up-and-Go” Test scores and 10-metre fast walking speed) in outcome measures in the full sample. After training, no between-group differences were observed in the tests.

**Conclusion:**

Our results indicate that game-based visual feedback with dynamic balance training has effects comparable to those of conventional balance training in patients with hemiplegia.

## LAY ABSTRACT

This study focused on the results of balance training with game-based real-time visual feedback for patients with hemiplegia compared with conventional training. The intervention group underwent training using the Physio Space™ system, which contains various game modules and provides visual cues to regulate posture in order to maintain balance. The main results were measured as the change in balance capacity and postural control. Walking ability and speed were also assessed. This study showed that dynamic standing balance training with game-based real-time visual feedback affects balance and gait, which are comparable to those of conventional balance training in patients with hemiplegia.

Patients with hemiplegia often experience gait deviation, plus difficulty with transfers and independent walking (1). Maintaining balance, a basic and important component of proper mobility, improves the confidence of these patients in participating in activities of daily living (2). Balance can be subcategorized into static and dynamic balance. Dynamic balance includes activities such as walking naturally, walking fast, and turning round, whereas static balance encompasses standing still or on a single leg. Dynamic balance control requires appropriate weight shifting from one side of the body to the other while maintaining the centre of mass within the limits of stability (3). Walking can be defined as a series of losses and recoveries of balance, as the centre of mass is alternately shifted from one side to the other, creating a single pattern (right-to-left) during the gait cycle (4). Static and dynamic balance control require the use of ankle and hip strategies when people are experiencing external disturbances. Weight shifting to the paretic limb is challenging for patients with hemiplegia; the centre of pressure measured under the nonparetic limb is greater than that under the paretic limb. Additionally, impairments of the hemiparetic side during forward weight shifting limit the motor system of patients with hemiplegia (5). The participation of the affected side in postural stabilization is limited, and the unaffected side cannot adequately compensate for the deficit on the affected side (6).

Vision is a crucial component of balance, serving as a key sensory input in the balance control system (7). It detects postural adaptations and provides information concerning the body’s orientation relative to the environment. For instance, movement within the visual field, such as a surrounding room, consistently evokes a self-motion illusion followed by a postural response (8). However, when an individual is provided with precise information on the timing and nature of the visual field’s motion, the postural response decreases (9). Additionally, the velocity of visual motion affects the magnitude of the evoked postural response; fast visual motion and eye movement is typically associated with the objects’ movement rather than self-motion (10, 11). After a stroke, visual impairment is seen in different groups of people, which encompasses visual field, the velocity of eye movement, and other ocular diseases (12, 13). All of these may have negative effects on functional recovery.

Currently there are many different types of rehabilitation training methods. Multi-Exergames were chosen for the training of balance, with the central pressure decreased gradually during the selected game (14). Commercial video games were used for balance training and there was improvement in the functional reaching test (15). Nintendo Wii Fit games are used more than other games for motor rehabilitation (16) and the Wii Fit game system stimulates people’s interest and improves the motor function and balance control (17). Games combined with circuit training exercise may improve balance, walking function, and quality of life of patients after stroke (18). Non-immersive virtual reality game-based training can improve dynamic balance and knee hyperextension control in chronic stroke (19).

The objectives of the present study were to (*i*) determine whether game-based real-time visual feedback combined with dynamic standing balance training is better than a conventional balance training programme, (*ii*) investigate whether game-based real-time visual feedback combined with dynamic standing balance training could improve standing balance in patients with hemiplegia.

## METHODS

### Trial design

This study was designed as a single-blind, randomized controlled trial. Participants were randomly assigned to either the experimental group (EG) or control group (CG), depending on the code within a sealed opaque envelope. The therapist responsible for outcome assessment was blinded to the group allocations of the participants. The study protocol was registered on the Chinese Clinical Trial Registry (ChiCTR2300067275, https://www.chictr.org.cn). All procedures adhered to the principles outlined in the Declaration of Helsinki. This study was approved by the ethics committee of Yangzhi Rehabilitation Hospital (Shanghai Sunshine Rehabilitation Center). All patients provided written informed consent before study enrolment.

### Participants

The study was conducted in Yangzhi Rehabilitation Hospital (Shanghai Sunshine Rehabilitation Center). We screened inpatients who were diagnosed with hemiplegia (disease duration: ≥ 1 month) from January 2023 to December 2023. The inclusion criteria were as follows: (*i*) hemiplegia caused by stroke, with brain lesions confirmed through computed tomography or magnetic resonance imaging; (*ii*) age 18–80 years; (*iii*) ability to stand for 20–30 min under supervision; and (*iv*) a Mini-Mental State Examination score of ≥ 20. The exclusion criteria were as follows: (*i*) comorbid diagnosis of cancer, tuberculosis, or severe osteoporosis; (*ii*) concurrent mental disease or cognitive impairment; (*iii*) severe cardiovascular or respiratory problems; and (*iv*) other serious conditions that would prevent a patient from completing the training programme.

### Participant flow

**Fig. 1:**
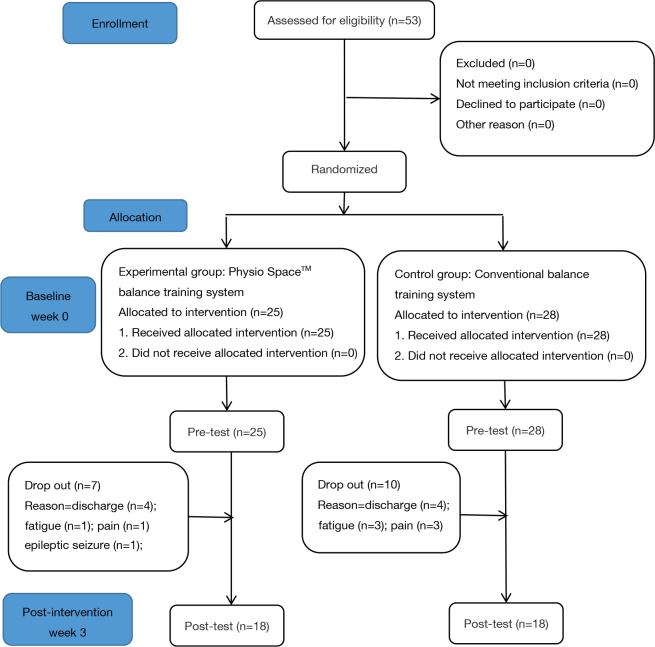
Participant flowchart. CONSORT diagram of this study: 53 patients with hemiplegia were assessed; all of them met the inclusion criteria. The patients were randomly divided into 2 groups (experimental group, *n* = 25; control group, *n* = 28). Patient attrition occurred in all groups, leaving 18 participants each in group.

During the recruitment period, 53 patients with hemiplegia were assessed (EG: *n* = 25, CG: *n* = 28), all of whom met the inclusion criteria. During the implementation of the experiment, 8 participants were excluded because they left the rehabilitation centre before completing the training protocol (EG: *n* = 4; CG: *n* = 4), 4 were excluded due to fatigue during the intervention and because they gave up the session (EG: *n* = 1; CG: *n* = 3), 4 were excluded due to excessive pain (EG: *n* = 1, CG: *n* = 3), and 1 was excluded owing to seizures that occurred during other treatments (EG: *n* = 1). Finally, 36 patients remained after the intervention; the 2 groups comprised 18 patients each (Fig. 1).

### Interventions

After recruitment, participants were randomly divided into 2 groups: the EG (*n* = 25), who underwent balance training with visual feedback using the Physio Space™ system, and the CG (*n* = 28), who underwent conventional training.

Patients in the CG completed a 3-week cycle of 20-min conventional rehabilitation therapy daily, 5 days per week. This conventional therapy involved standing naturally while shifting their weight forwards to backwards and left to right (5 min), pointing their finger as far as possible in different directions without stepping (5 min), standing on a soft surface (such as a soft mat) (5 min), and standing on a soft mat with eyes opened and closed (5 min). Participants could regulate their posture and standing balance independently. The difficulty of the exercises increased according to the patient’s self-assessment and concurrent approval by the physiotherapist. If participants are able to finish most training sequences (first stage), the conventional therapy may be designed as standing normally, catching and throwing the ball randomly (5 min); standing unsupported with 1 foot directly in front of the other (5 min); standing on a soft surface (such as a soft mat), catching and throwing ball randomly (5 min); standing without support with a single leg as long as the patients can do so (5 min).

**Fig. 2:**
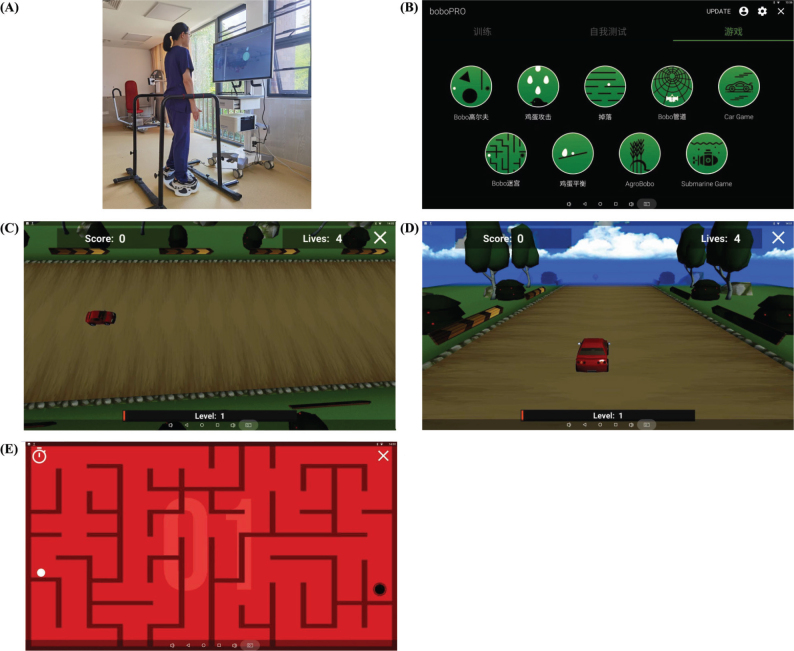
Physio Space™ balance system with a balancing platform. (A) Physio Space™ balance system configuration (a non-patient model). (B) User interface to select among 9 game modules. (C) Weight shift from forwards to backwards. (D) Weight shift from right to left. (E) Weight shifts in a variety of directions.

Patients in the EG completed a 3-week cycle of 20-min sessions with the Physio Space™ balance system daily for 5 days per week. The Physio Space™ hardware comprises a soft mat, touch screen, and 3-axis balancing platform; the software operates the dynamic balance training programme and provides visual feedback during the entire session. The programme contains 9 game modules that direct the user to shift weight from right to left, from front to back, and in a variety of other directions, depending on the patient’s ability (Fig. 2B). The soft mat was placed on the floor, the Physio Space™ balancing platform was placed on the soft mat, and patients standing on the platform kept their arms at their sides naturally (Fig. 2A). Subsequently, according to patients’ interests, they should choose 3 games from 9 game modules; these games stimulated patients’ weight shifting. The training sequence was forwards to backwards (Fig. 2C), left to right (Fig. 2D), and in a variety of directions (Fig. 2E). Then, they were required to finish the 3 games twice, each within 20 min. The difficulty of the exercises increased at a pace according to the patient’s self-assessment and concurrent approval by the physiotherapist; if a patient could complete a game with 80% accuracy, the patient was allowed to attempt the subsequent difficulty level. Interventions in both CG and EG were performed by a certified physiotherapist experienced in computerized balance training, and the physiotherapist provided supervision and security.

### Outcome measures

The primary outcome measures included the Functional Reaching Test (FRT) and Berg Balance Scale (BBS) as measures of balance capacity, whereas the secondary outcomes focused on walking ability, which involved the Timed “Up-and-Go” (TUG) test and 10-m walk test. All assessments were performed by the same therapist, who was blinded to the group assignment, and EG and CG measurements were performed twice; first time after recruitment and before intervention, and second time after 3 weeks’ intervention.

*Functional Reaching Test.* The FRT is a quick and easy method for assessing dynamic standing balance and for determining a patient’s risk of falling (20). From the standing position, patients were asked to reach forward to a point on a wall at shoulder height as far as they could without taking a step, and touch the wall. The variable of interest was the average maximum reaching distance across 3 trials.

*The BBS.* The BBS is a 14-item test designed to measure dynamic and static balance. For static balance, the body’s centre of gravity was maintained within the base of support. For dynamic balance, the centre of gravity was maintained within the base support while in movement (21). The BBS demonstrated good intra- and inter-rater validity and reliability in clinical assessments (22, 23). Scores ranged from 0 to 56, with scores < 40 indicating a risk of falling (24).

*The TUG.* The TUG test is a common measure used to determine the risk of falling in clinical assessments (25). Patients were asked to rise from a standard armchair, walk to a marker placed 3 m away at their preferred pace, turn, walk back, and sit down again. They were allowed to practise before commencing the timed test.

*10-m walk test.* The patients were asked to walk 10 m independently. The patients were timed over the middle 6 m and the scores for normal (nVel) and maximum (fVel) walking speeds were recorded (26, 27).

### Statistical analysis

All statistical tests were performed using SPSS software (version 26.0; IBM Corp, Armonk, NY, USA). Values were compared between the EG and CG before and after the intervention using a two-way repeated-measures analysis of variance (ANOVA). Differences in categorical variables were analysed using independent *t*-tests and χ^2^ tests. For all tests, *p* < 0.05 was considered significant.

## RESULTS

### Baseline data

The demographic and experimental baseline characteristics of the participants are presented in Table I. No significant differences were observed between groups in terms of demographic or health variables.

**Table I:** Demographic, health, and testing data of patients at baseline

Sample features	EG (*n* = 18)	CG (*n* = 18)	*p*-value
Age (years)	49 ± 18.41	51.11 ± 14.74	0.71
Sex (M/F)	15/3	18/0	NT
Side (right/left)	12/6	11/7	NT
Height (cm)	171.83 ± 9.46	173.61 ± 6.28	0.51
Weight (kg)	72.94 ± 17.86	75.44 ± 11.05	0.62
Time since stroke (month)	14.06 ± 17.60	18.39 ± 16.41	0.45
BBS	48.83 ± 7.35	44.33 ± 11.04	0.16
FR (cm)	26.44 ± 7.29	23.94 ± 7.73	0.33
nVel (m/s)	0.61 ± 0.50	0.56 ± 0.51	0.74
fVel (m/s)	0.83 ± 0.71	0.67 ± 0.59	0.45
TUG (s)	25.06 ± 15.08	37 ± 28.18	0.12

Values are expressed as mean±standard deviation.

BBS: Berg Balance Scale; CG: control group; EG: experimental group; FR: Functional Reaching Test; nVel: normal velocity; fVel: fastest velocity; TUG: Timed “Up-and-Go” test; M: male; F: female; NT: not tested.

### Outcomes

The results of the post-training assessments are presented in Table II. Repeated-measures ANOVA showed that across the full sample, both in the EG and CG, patients showed improvement in the BBS score (F = 30.888 *p* < 0.05), FR score (F = 6.729 *p* = 0.014), fVel (F = 6.955 *p* = 0.013), and TUG score (F = 23.573 *p* < 0.05) 3 weeks after the intervention. No change was observed in nVel in the full post-intervention sample. However, there were no significant differences observed in changeable variables between the EG and CG after the 3-week intervention.

**Table II:** Pre- and post-intervention assessment scores (mean ± SD) of the 2 study groups

Evaluation tools	Study group		Analysis of covariance					
	EG (*n* = 18)	CG (*n* = 18)	Group effect		Time effect		Group*Time	
	Week 3	Week 3	F	*p*-value	F	*p*-value	F	*p*-value
BBS	52.44 ± 5.31	48.89 ± 7.30	2.46	0.13	30.89	< 0.05	0.41	0.53
FR (cm)	29.06 ± 8.73	25.06 ± 6.52	1.79	0.19	6.73	0.01	1.09	0.30
nVel (m/s)	0.67 ± 0.49	0.56 ± 0.51	0.26	0.62	1.00	0.32	1.00	0.32
fVel (m/s)	1.06 ± 0.73	0.78 ± 0.65	1.08	0.31	6.96	0.01	0.77	0.39
TUG (s)	20.22 ± 13.40	33.17 ± 27.70	2.87	0.10	23.57	< 0.05	0.31	0.58

SD: standard deviation; CG: control group; EG: experimental group; BBS: Berg Balance Scale; FR: Functional Reaching Test; nVel: normal velocity; fVel: fastest velocity; TUG: Timed “Up-and-Go” test.

## DISCUSSION

The primary purpose of this study was to determine whether game-based real-time visual feedback combined with dynamic standing balance training was better than conventional balance training programmes. However, no significant differences were observed in the balance and gait parameters between training with real-time visual feedback combined with dynamic balance and conventional balance training. This finding indicates that dynamic balance training with real-time visual feedback is not superior to conventional physical therapy.

The secondary purpose of this study was to investigate whether game-based real-time visual feedback combined with dynamic standing balance training can improve the standing balance in patients with hemiplegia. Based on the results of this study, in both EG and CG, the outcomes of balance (BBS, FR) and gait (TUG, fastest velocity) were all improved after a 3-week intervention; that is, the ability to maintain balance and walk were all improved in terms of static and dynamic balance, walking velocity, and reduced risk of falling.

After a 3-week intervention, the outcome measurements of static (BBS, FR) and dynamic balance (TUG, fastest velocity) were improved in the EG, suggesting that the game-based real-time visual feedback combined with dynamic standing balance training can improve the balance control and walking ability in patients with hemiplegia. The game system provides an unstable standing platform for participants, which may stimulate ankle and hip balance strategies. These strategies are fundamental for maintaining both static and dynamic balance. The training method requires participants to move forwards and backwards, stimulating ankle plantarflexion and dorsiflexion, as well as hip flexion and extension (28). Moving left and right involves ankle inversion and eversion, as well as hip abduction and adduction (29). Controlling the range of motion, muscle activation, torque, and power of the hip and ankle is crucial for effective balance control and the gait cycle (4).

The chosen game module requires participants to shift their weight from forwards to backwards, from left to right, and in a variety of other directions. This direct method of training weight shifting can significantly enhance balance abilities. Well-targeted exercise therapy programmes that focus on balance, weight-shifting, and gait training have been shown to improve postural control in the chronic phase after a stroke (30). A new weight-shifting-based robot control system has effectively improved the weight-bearing amount and weight-bearing ratio on the operated side shortly after total hip arthroplasty, enhancing static standing postural control (31). Additionally, a smartphone-based wearable telerehabilitation system for home use focuses on training dynamic weight-shifting balance exercises for patients with Parkinson’s disease, providing significant benefits for their daily exercise routines and allowing for off-site therapist support (32). Therefore, weight-shifting exercises are a direct and effective way to train balance and walking ability.

The motion targets and game task in the game modules provide continuous visual motion and changing visual fields (see Figs 2D and E). Contrast sensitivity, motion detection, and the size of the visual field can affect functional behaviours, visual processing, and fall risk. The illusion of self-motion in response to visual motion is primarily influenced by stimuli in the peripheral visual field. Optic flow can produce illusions of self-motion, disturbing upright balance during both standing and walking. Detecting self-motion from optical flow is crucial for balance control (33). Peripheral vision plays a more significant role in back-and-forth body oscillation compared with central vision (34). Additionally, the visual component acts as a compensatory mechanism for postural stability, especially when proprioceptive input is inadequate (35). Therefore, incorporating changeable visual motion and various visual fields in training can improve postural control and self-motion perception, and reduce the risk of falls.

During the intervention, participants were required to control the balance board and navigate through the game process. Each game incorporates mechanisms of “reward” (diamonds) and “punishment” (bombs). Participants must manoeuvre the balance board (depicted as a car in Figs 2D and E), swiftly accepting rewards and avoiding punishments without prior cues appearing. This necessitates quick reactions to maintain balance and respond promptly to the appearance of targets on the screen. Fast reaction speed is crucial because it correlates with better balance performance, whereas a longer reaction time is linked to poorer postural stability (36). For older adults, reducing the reaction time can potentially mitigate instability and enhance their lower-limb balance and strength, thereby improving their overall quality of life (37).

During clinical training, therapists can choose either balance training combined with game-based and visual feedback or conventional balance training. Game-based balance training may offer different modules for patients and can further complicate training only gradually; however, it can effectively relieve depression and improve mental health in patients with hemiplegia, and may positively impact cognitive performance, mobility, and emotional state (38). The disadvantage of game-based balance training is that it depends on the software and hardware support of the training section. The advantage of conventional therapy is convenience and the physical therapist can have additional designs of training sequences and methods depending on the performance of patients during daily therapy. However, the disadvantage is that there are few influences except motor function, and it cannot supply the objective result of patient improvement.

### Limitations

This study has some limitations. First, the sample size was small for both the CG and EG patient cohorts and there were only 3 females in the study. Therefore, the results may not be generalizable to a wider population of patients with hemiplegia. Accordingly, studies with relatively large sample sizes should be conducted to investigate the role of demographic factors. Second, the study protocol was retrospective registration. Furthermore, questionnaires or scales could be used to determine the participants’ motivation, confidence, and cognitive ability (such as attention), all of which may provide other explanations. Meanwhile, follow-up is needed to facilitate data collection for long-term influence.

### Conclusion

The Physio Space™ balance system, which combines dynamic standing balance training with game-based real-time visual feedback, has effects on balance and gait that are comparable to those of conventional balance training in patients with hemiplegia.
